# Computational Study on Subdural Cortical Stimulation - The Influence of the Head Geometry, Anisotropic Conductivity, and Electrode Configuration

**DOI:** 10.1371/journal.pone.0108028

**Published:** 2014-09-17

**Authors:** Donghyeon Kim, Hyeon Seo, Hyoung-Ihl Kim, Sung Chan Jun

**Affiliations:** 1 School of Information and Communications, Gwangju Institute of Science and Technology, Gwangju, South Korea; 2 Department of Medical System Engineering, Gwangju Institute of Science and Technology, Gwangju, South Korea; SUNY Downstate MC, United States of America

## Abstract

Subdural cortical stimulation (SuCS) is a method used to inject electrical current through electrodes beneath the dura mater, and is known to be useful in treating brain disorders. However, precisely how SuCS must be applied to yield the most effective results has rarely been investigated. For this purpose, we developed a three-dimensional computational model that represents an anatomically realistic brain model including an upper chest. With this computational model, we investigated the influence of stimulation amplitudes, electrode configurations (single or paddle-array), and white matter conductivities (isotropy or anisotropy). Further, the effects of stimulation were compared with two other computational models, including an anatomically realistic brain-only model and the simplified extruded slab model representing the precentral gyrus area. The results of voltage stimulation suggested that there was a synergistic effect with the paddle-array due to the use of multiple electrodes; however, a single electrode was more efficient with current stimulation. The conventional model (simplified extruded slab) far overestimated the effects of stimulation with both voltage and current by comparison to our proposed realistic upper body model. However, the realistic upper body and full brain-only models demonstrated similar stimulation effects. In our investigation of the influence of anisotropic conductivity, model with a fixed ratio (1∶10) anisotropic conductivity yielded deeper penetration depths and larger extents of stimulation than others. However, isotropic and anisotropic models with fixed ratios (1∶2, 1∶5) yielded similar stimulation effects. Lastly, whether the reference electrode was located on the right or left chest had no substantial effects on stimulation.

## Introduction

Electrical neuromodulation has long been used to relieve neurological disorders, including: essential tremor [1], [2]; chronic stroke [3]; chronic pain [4]; Parkinson’s disease [2], [5]; movement disorder [6]; refractory epilepsy [7]; depression [8]; aphasia [9], and dystonia [10]. Electrical neuromodulation may be classified either as noninvasive or invasive depending on the method used to deliver the electrical current to the target brain structures. Noninvasive cortical stimulation methods, such as transcranial magnetic stimulation (TMS) [11] and transcranial direct current stimulation (tDCS) [12] can excite underlying neural structures without incising the skin or opening the cranium. These have been used extensively to investigate the excitability of the brain, enhance brain plasticity responsible for recovery from brain injuries, and modulate cortical or oscillatory activities in cortico-subcortical networks [13]–[17]. However, these noninvasive methods of cortical stimulation have several limitations, including less focused stimulation, difficulty of concurrent rehabilitative training, necessity of the presence of medical personnel to manipulate the devices, and frequent visits to the hospital. In parallel with the use of noninvasive brain stimulation techniques, invasive methods, in which electrical current is delivered to the brain through implanted electrodes (deep brain stimulation: DBS), or on the cortex (cortical stimulation) have been used to treat patients with Parkinson’s disease, epilepsy, and stroke [3], [18], [19]. Despite their invasiveness, these methods have advantages in providing cortical stimulation and concurrent rehabilitative training.

Cortical stimulation consists of epidural cortical stimulation (ECS) or subdural cortical stimulation (SuCS), depending upon whether the electrodes are placed epidurally or sudurally. Due to safety issues (seizures [20]), ECS has been performed widely in clinical surgery for cortical stimulation [6], [8], [9], [21], [22]. For this reason, the effects of ECS have been investigated by computational studies and clinical trials [6], [8], [23]–[27]. However, in some cases, if brain atrophy–which is observed frequently in candidates for ECS–is moderate or severe, the cerebrospinal fluid-filled space can be a barrier to delivery of electrical stimulation to the cortex despite its electrical conductance. Therefore, the stimulation effects of ECS are influenced by the thickness and electrical properties of both dura mater and cerebrospinal fluid [24], [25]. Recently, due to diminished stimulation effects, the electrode was repositioned from the epidural to subdural space in a patient who had ECS surgery [4]. In this case, SuCS can be an alternative therapy; for example, successful unilateral subdural motor cortex stimulation has been reported recently in a tremor patient [1]. For these reasons, extensive study of the effects of SuCS is needed.

A computational study may provide a better understanding of the mechanism of SuCS by estimating the effects of, and appropriate parameters for, stimulation (amplitude, frequency, electrode position, and electrode configuration) with the implicit assumption that the excitability of a neuron is linearly proportional to the magnitude of the current density (or electric field) in the brain [28], [29]. Computational studies of ECS have been conducted with a simplified extruded slab model of the precentral gyrus [23]–[26]. Using these models, they have revealed how electric current generated by brain stimulation is distributed in the brain. Furthermore, neuronal response has been predicted using computational models of epidural motor cortex stimulation [23], [24], [26]. Recently, our group developed a computational extruded slab model for SuCS [30], [31] and analyzed the variation in the amount of current flow in the brain when stimulation amplitude, electrode shape, and electrode position were altered. Thus far, these studies have analyzed the distribution of current density only around the precentral gyrus. This is beneficial, in that these models show the spatial distribution of current density around the target area in greater detail. However, SuCS can inject a higher current into the brain than can transcranial direct current stimulation (tDCS), because the electrode(s) for SuCS is (are) located directly above the cortex. Consequently, SuCS may have a much wider and deeper stimulation effect than tDCS or other non-invasive brain stimulation methods. Furthermore, using simplified brain models to estimate the distribution of current density induced by injected current or voltage may result in unreasonable predictions of the stimulation effect. Hence, to avoid such possible erroneous interpretations, it is essential to investigate the effects of a model of the head. To date, there have been lacking in invasive brain stimulation studies on how simplified brain models and realistic full brain models differ. Thus, in this work, we developed a realistic upper body model that consisted of a combined realistic brain model and simple chest model. This upper body model is more advantageous, in that it reflects the clinical situation in which neural stimulators are often placed on the chest together with electrodes in the brain. Further, it may reduce the non-negligible computational error that originates from substantial model mismatch, and enable us to understand the behavior of current density distribution induced by electrical stimulation to the whole upper body, as well as the whole brain. We expect that a clearer understanding of, and more refined parameters for, SuCS might result from this work, and reduce the risk of various side effects attributable to the use of SuCS with inaccurate parameters. In this particular study, we focused on paddle-array and single electrode stimulations with the disc electrode configurations that are in wide use.

## Methods

### MRI data and segmentation

**Figure 1:**
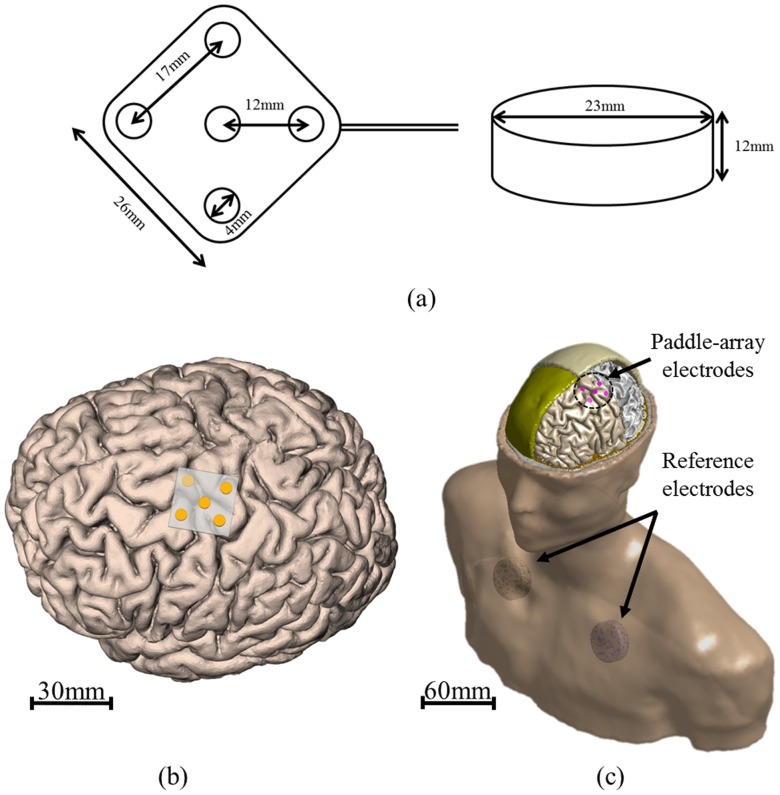
Specification of paddle-array and reference electrode and illustration of the paddle-array in the brain. The shape of a paddle-array having 5 metal electrodes and reference electrode (a). All metal electrodes of the paddle-array are disc type with a diameter of 4 mm. Also, only the contact side of the electrode (subsurface) is exposed. Illustration of the paddle-array on the gray matter in the brain (b) and our 3D computational model including the chest (c).

To generate a realistic brain model that includes the upper chest, we used two different sets of MRI data in this work: a brain MRI and a whole human body MRI acquired from SimNIBS [32] and the Visible Human Project of Korea [33]. We note that these human MRI data are anonymized, de-identified, and are publicly accessible. For this reason, the institutional review board (IRB) approval of Gwangju Institute of Science and Technology (GIST) was not required for this study. In brain modeling, the resolution of the brain model depends primarily on the resolution of the brain MRI data and MR sequence. Without fat suppression, the positions of the spongy bone and subcutaneous fat are displaced in the MR image due to a chemical shift artifact. This can cause a slight overlap between the brain and spongy bone, thereby reducing the accuracy of the skull reconstruction. To overcome this problem, we used four types of brain MRI data with 1 mm^3^ spatial resolution in SimNIBS: T1, T1 fat suppression, T2, and T2 fat suppression. The T1 image was used for cortical segmentation; the fat-suppressed T1 and T2 images were used to reconstruct the inner skull boundary, and the normal T1 and T2 images were used to reconstruct the outer skull boundary and the scalp (skin) surface. In processing the brain MRI data, we used FreeSurfer [34] for cortical segmentations, such as gray matter, white matter, ventricles, and cerebellum. Furthermore, we used the brain extraction tool, BET2 [35], for scalp and skull segmentation. In the whole body MRI, we simply extracted the shape of the upper body. For this, we employed the histogram shape-based image thresholding algorithm, Otsu’s method [36], using Seg3D (http://www.seg3d.org). Because the segmented brain and segmented upper chest are not matched spatially, spatial co-registration was performed manually by matching the bottom surface of the brain and the upper surface of the neck as closely as possible (Figure 1. b and c).

### Volume mesh generation

Based on the segmented data, using FreeSurfer [34] we generated surface meshes for each segmented tissue layer, such as gray matter, white matter, cerebrospinal fluid, ventricle, cerebellum, skull, and scalp. To generate the computational model for the SuCS, the anode electrode(s)’ surface just beneath the dura mater and the reference electrode’s surface implanted in the chest were incorporated into the whole mesh generation procedure. Intersection between adjacent surfaces (i.e., between gray matter and white matter) was not considered here; thus, intersection removal processing via Meshfix [37] was conducted. There was no model element with a mixture of conductivities to represent interfaces. The anode electrode(s) was (were) positioned on the hand area of the precentral motor cortex. In generating the mesh incorporating attached electrode(s) into the model, the shape of an electrode may be distorted easily. To minimize this problem, we merged the surface of electrode(s) and gray matter surface in the following way:

- Triangular elements on the gray matter surface that intersects with the electrode(s)’ surface were detected and removed.
- Then placed the electrode(s)’ surface on the gray matter surface.
- Finally filled the gap between the electrode(s)’ surface and that of the gray matter with new triangular elements.

**Figure 2:**
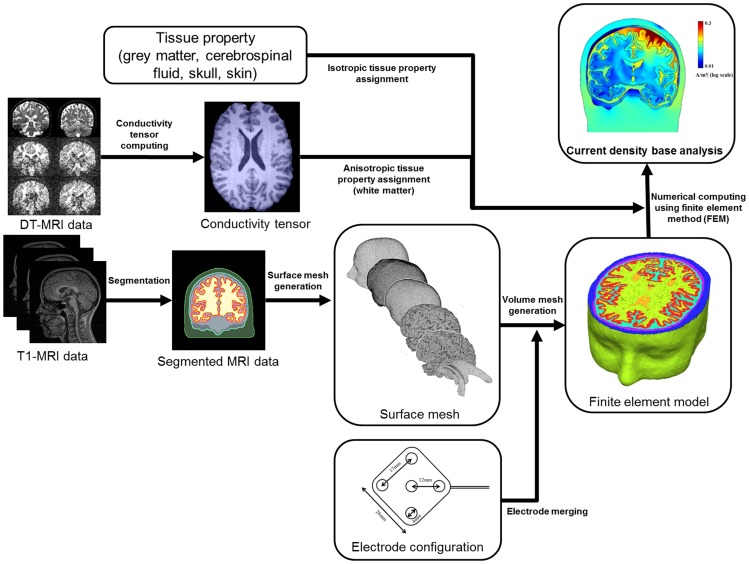
Diagram describing the procedure of volume mesh generation.

Our mesh generation strategy is depicted in Figure 2. In this study, based on this intersection-free surface mesh, we generated a volume mesh consisting of 7.3 million tetrahedrons using TetGen [38], iso2mesh [39], and matlab.

### Electrode configuration

To consider clinical situations [1], [3], [22], we used a paddle-array for anodal stimulation, as illustrated in Figure 1a. This paddle-array configuration was designed for cortical stimulation and has been used to treat stroke patients [3]. Because we focused on unipolar, subdural motor cortex stimulation, a cylinder-shaped reference electrode was modeled and implanted in the chest. For the paddle-array stimulation, all five electrodes were used to input the given voltage, and one electrode in the middle of the paddle was used for the single electrode stimulation. In tDCS, it has been reported that the positions of the anode or cathode electrode and the reference electrode change the distribution of the electric field in the brain [13]. In this work, to investigate the current density distribution behavior across varying positions of the reference electrode, two representative positions for the reference electrode (left or right side of the chest) were used. Figures 1b and c illustrate an implanted paddle-array on the precentral gyral region and an attached reference electrode on the chest, respectively.

### Electrical conductivity distribution

In reality, the electrical conductivity of the head or body is quite different over tissue layers or organs such as scalp, skull, CSF, dura mater, gray matter, white matter, skin, lung, liver, heart, bone, and so on. For its simplicity, such variation in conductivity is accounted for in the computational model by assigning uniform (isotropic) conductivity distributions to each tissue layer, as follows: white matter (0.126 S/m); skull (0.01 S/m), and scalp and body (0.465 S/m) [13]. Particularly, for assigning the conductivity of body, we simplified the body’s conductivity as skin’s conductivity. Even the body (chest) consists of many types of tissues [40], conductivities of most tissues are not big different from skin’s conductivity except for bone and lung. In addition, we noted that in voltage and current stimulations the body modeling with more detailed conductivities of most tissues yielded no significant difference from our body modeling with skin conductivity only (not shown here).

Further, as it is known that most current is likely to flow along the fiber in the white matter, the distribution of anisotropic conductivity in the white matter was considered using diffusion tensor imaging (DTI). To estimate the anisotropic conductivity tensor in the white matter, we used the general assumption that the eigenvector of the diffusion tensor is the same as the conductivity tensor [41]. Then, conductivity tensor can be expressed by known eigenvector (S: orthogonal matrix consisting of unit length eigenvectors) and unknown eigenvalue (σ^trans^: transverse direction, σ^long^: longitudinal direction, equation 1). To estimate the eigenvector of the conductivity tensor, we used a direct approach with the volume constraint method [42] (σ_nor_vi_: normalized eigenvalues, σ_vi_: diffusion tensor eigenvalue, σ_iso_: isotropic white matter conductivity, equation 2) and fixed ratio method (r: ratio coefficient, equation 3). The isotropic conductivity, normalized anisotropic conductivity, and various fixed ratios (anisotropic factor r = 2 if 1∶2, r = 5 if 1∶5, r = 10 if 1∶10) of anisotropic conductivity, such as 1∶2, 1∶5, 1∶10, were considered for the investigation of the comparative stimulation effects of both isotropic and anisotropic conductivity distributions. The degree of anisotropy in the white matter can be described by fractional anisotropy (FA, λ_n_: n-th eigenvalue of conductivity tensor, equation 4).
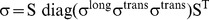
(1)




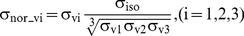
(2)




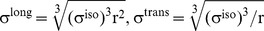
(3)




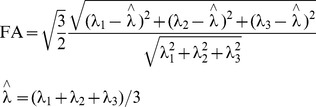
(4)


### Computational model for SuCS

Based on the 3D human MRI data and anatomical parameters, we developed a realistic upper body model composed of head and chest, which is considered the base model in this work (details below). In addition, we investigated how stimulation effects would differ between a whole brain model and a partial brain model; thus, we wanted to determine whether a partial brain model (widely used in this field) is effective in understanding the mechanisms of cortical stimulation. For this purpose, we generated the following computational models:

▪ Realistic upper body model (base model)

**Figure 3:**
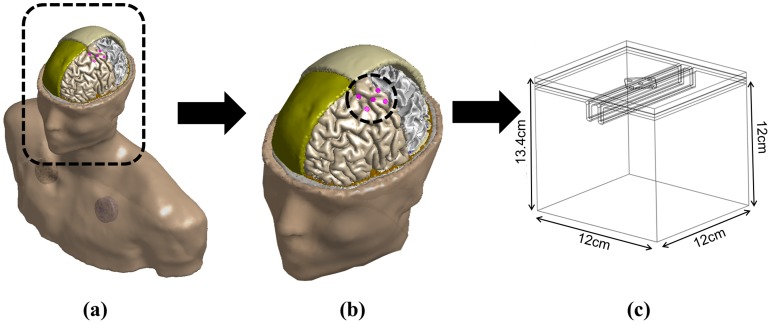
Geometric structure of the brain model. A realistic upper body model (a), realistic full brain-only model (b), and simplified extruded slab model (c).

This is the most realistic model in this work; it is advantageous, in that a more realistic consideration of the reference electrode and its positioning on the chest is possible. This model is illustrated in Figure 3(a). With this model we investigated current density distributions in the following comparative studies:

  - electrode configuration: paddle-array and single electrode stimulation
  - conductivity: isotropic and anisotropic conductivities of white matter
  - reference electrode positioning: right or left chest reference electrode positions

▪ Realistic full brain-only model

This model considers only the head (full brain) and part of the neck, and is generated by truncating a realistic upper body model at the neck. The bottom surface of the neck was grounded to yield the effect of a reference electrode, as a realistic reference electrode cannot be considered in this model. With this model, we investigated to see if the realistic full brain-only model is good enough to estimate stimulation effects, comparing to the realistic upper body model. An illustration of this model is shown in Figure 3(b).

▪ Simplified extruded slab model (partial brain model)

This is an extruded slab model representing the area around the precentral gyrus in the brain [30]. This simplified model was generated from anatomical parameters (widths of gyrus and sulcus, etc.) and only represents the part of the brain near the motor cortex. To take into account the reference electrode into which current flows as a sink, the side and bottom surfaces of the model were grounded, with the voltage on these surfaces set to 0. Figure 3(c) outlines this model. We note that this simplified model is used commonly in computational studies of cortical stimulation [23]–[26], [30], [31], [43].

### Mathematical Formulation for Voltage and Current Stimulation

In general, cortical stimulation involves injecting direct electrical current or induced current by voltage into the brain via electrode(s). Maxwell’s equation explains such electrical behavior within the brain; thus, the following Laplace equation is governed in the model Ω:
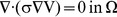
(5)




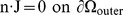





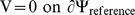





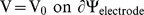



Here V and σ are an electrical potential and a conductivity distribution in Ω, respectively. n and J are the normal vector to the boundary and the current density, respectively. V_0_ is considered as an input voltage value. Assuming that the electric flux through the skin (boundary of the model) is negligibly small (that is, insulated), the Neumann boundary condition is applied to the outer boundaries of the model, such as the scalp, skin of the neck and chest, and the bottom of the chest. In general, current mainly flows along the outer surface of the model, so the orthogonal current flow to the outer surface should be zero, which is mathematically presented as Neumann boundary condition (2^nd^ equation in equation (5)). Further, the Dirichlet boundary conditions are applied at the reference electrode surface in the model and at the upper boundary on the electrode(s)’ surface.

Our mathematical approach was formulated for voltage stimulation. However, current stimulation is more mainly used in clinical or experimental practice than voltage stimulation. For this reason, the conversion of voltage stimulation into current stimulation may be helpful in understanding the stimulation effects in a quantitative way. To convert voltage stimulation into current stimulation, model impedance is required. From voltage stimulation, we estimated the total output current yielded by a given model with an input voltage of 1V. Next, model impedance was estimated according to Ohm’s law: voltage (V) = output current (A) × impedance (Ω). The estimated impedances for each model are tabulated in Table 1.

**Table 1:** Impedance and output current of each model.

Brain model type	Electric property	Electrode configuration	Impedance (Ω)	Output current (mA)
Realistic upper body	Isotropic	Paddle-array	136.4	7.33
Realistic upper body	Anisotropic (nor)	Paddle-array	136.7	7.31
Realistic upper body	Anisotropic (1∶2)	Paddle-array	139.5	7.17
Realistic upper body	Anisotropic (1∶5)	Paddle-array	142.2	7.03
Realistic upper body	Anisotropic (1∶10)	Paddle-array	138.3	7.23
Realistic upper body	Isotropic	Single electrode	348.4	2.87
Realistic upper body	Anisotropic (nor)	Single electrode	348.4	2.87
Realistic upper body	Anisotropic (1∶2)	Single electrode	352.1	2.84
Realistic upper body	Anisotropic (1∶5)	Single electrode	354.6	2.82
Realistic upper body	Anisotropic (1∶10)	Single electrode	349.7	2.86
Realistic brain-only	Isotropic	Paddle-array	110.5	9.05
Realistic brain-only	Anisotropic(nor)	Paddle-array	110.9	9.02
Realistic brain-only	Isotropic	Single electrode	327.9	3.05
Realistic brain-only	Anisotropic(nor)	Single electrode	327.9	3.05
Simplified extruded slab	Isotropic	Paddle-array	111.7	8.95
Simplified extruded slab	Anisotropic(nor)	Paddle-array	89.9	11.12
Simplified extruded slab	Isotropic	Single electrode	467.3	2.70
Simplified extruded slab	Anisotropic(nor)	Single electrode	347.2	2.88

For this numerical problem (equation (5)), we introduced the finite element method (FEM). To solve the boundary value problem with the FEM, volume mesh (consisting of tetrahedral elements) was generated in an adaptive fashion; we applied a volume constraint factor to each model component, so that the mesh was coarse around simple structures, and finer around complex structures. Considering the computational load and accuracy, a volume mesh composed of about 7.3 million elements was used in this work. Element volume ranged from 2.2×10^−8^ to 1.0×10^1^ mm^3^ (averaged element volume: 1.2 mm^3^). To ensure the convergence of this FEM analysis, computation on a finer mesh (about 11.6 million elements) was also performed, and we found that it was not substantially different from our base model (maximum relative difference in voltage values over the whole computational domain was less than 2.6%). Here, we varied input voltage from 0.5 V to 5.0 V in increments of 0.5 V. All simulations were conducted in COMSOL Multiphysics 4.3b. The total computation time for each model was approximately 4 hours on a personal computer (i7 Quadcore 3.6 GHz with 32 GB RAM).

### Quantitative analysis

In SuCS, anode or cathode electrodes generate extracellular current flow inside the brain, which changes the trans-membrane voltage of neurons [25], [26], [44], [45], thereby likely triggering action potentials. To investigate the effect of cortical stimulation in a quantitative manner, it is common to measure the volume or penetration depth of the region where neurons likely to be triggered passively by current injection are located. For this purpose, the minimum current density needed to evoke neuron excitation in the motor cortex due to passive stimulation should be determined. Such a value is called the “motor current density threshold” (MCT). In this study, the MCT was set at 2.5 A/m^2^ at 50 Hz, which has been reported in the literature and examined clinically [46]. Our proposed MCT threshold is the value estimated by TMS. Basic underlying physics (injecting regulated current into the brain) between TMS and cortical stimulation are the same on the scalp and cortex, except where magnetic or electrical stimulation is regulated. Thus, our MCT threshold may be sufficient for our purposes; although validation of this argument would be beneficial, it was beyond the scope of this work.

Based on the MCT, two efficiency measures quantifying the stimulation effect were considered here, as follows [30], [31]:

▪ Effective volume

The effective volume is defined as the volume of the region that has a magnitude of current density over the MCT. This is believed to represent the extent of the gray matter and white matter that experiences neural excitation.

▪ Effective depth of penetration

The effective depth of penetration is defined as the diameter of the region that has a magnitude of current density over the MCT. This diameter is measured from the center of the electrode (the middle electrode in the paddle-array) along the line perpendicular to the electrode surface.

It is known that MCT may vary depending on the cortical region (e.g., gray matter or white matter) because the majority of neuronal types (axons, dendrites, soma) and their orientations vary depending upon the cortical region [23], [24], [26], [44], [45]. However, this was not considered here and for our comparisons, the same threshold was used for the whole brain.

## Results

### Stimulation effects between paddle-array and single electrode

**Figure 4:**
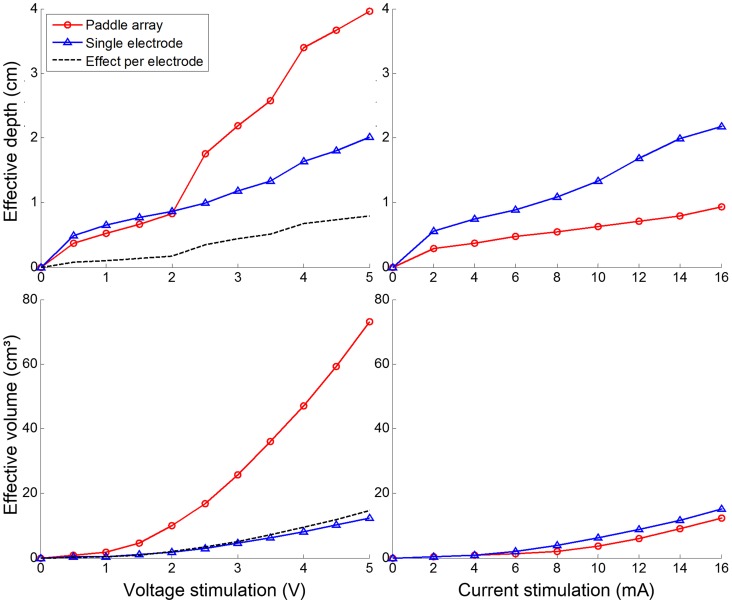
Effect of types of stimulation electrodes on variation of the effective depth of penetration and effective volume with stimulation voltage (V) and current (mA). Paddle-array and single electrode stimulations are compared quantitatively. Paddle-array (effect per electrode) is calculated using the effective depth of penetration and volume for the paddle-array divided by the number of electrodes (5).

**Figure 5:**
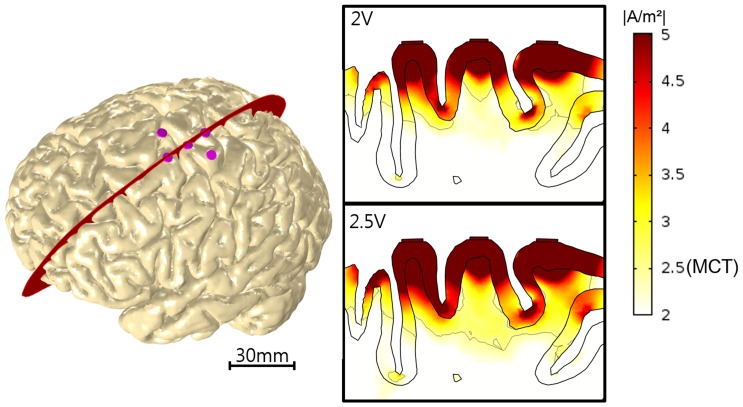
Current density distribution in the brain. A 3D visualization of the brain with implanted paddle-array (left). The red line is the tangential slice of the right top and right bottom electrodes. The current density map is of the tangential slice (log scale) in the brain. We visualized only over the motor cortex threshold (2.5 A/m^2^); the thin black line is the edge of the threshold.

We investigated how electrode configurations (paddle-array and single electrode) influence stimulation effects. The stimulation effects (the effective penetration depth (cm) and volume (cm^3^)) of the two electrode types over varying stimulation voltages (from 0.5 to 5 V in increments of 0.5 V) and currents (from 1 to 16 mA in increments of 2 mA) are presented in Figure 4. For the voltage stimulation in the paddle-array, the effective depth of penetration varied nonlinearly from 0.4 cm (at 0.5 V) to 4 cm (at 5.0 V). For the voltage stimulation in the single electrode, the depth ranged almost linearly from 0.4 cm to 2 cm. Interestingly, for an input voltage of 2 V or fewer, the single electrode voltage stimulation yielded comparable penetration depth to the paddle-array, while the paddle-array voltage stimulation showed substantially higher penetration depth at an input voltage of 2.5 V or greater. When the effective penetration depth per electrode was taken into account for the paddle-array, the paddle-array voltage stimulation appeared to be slightly less efficient than the single electrode voltage stimulation in terms of the contribution to penetration depth. Interestingly, the effective depth increased rapidly between 2 V and 4 V inputs with the paddle-array voltage stimulation. It should be noted that the 2 V stimulation input behaved like a threshold of input voltage for deep penetration of current. When input stimulation was 2 V, current penetrated into the gray matter and shallow layer of the white matter. Above 2 V, the current penetrated into much deeper layers of the white matter, as illustrated in Figure 5.

Furthermore, the effective volume ranged from 0.78 cm^3^ (at 0.5 V) to 73.1 cm^3^ (at 5.0 V, total volume) in the paddle-array voltage stimulation. In the single electrode voltage stimulation, as well, the volume ranged from 0.24 cm^3^ (at 0.5 V, total volume) to 12.3 cm^3^ (at 5.0 V, total volume). Similar to the effective depth of penetration, when the effective volume per electrode was taken into account in the paddle-array voltage stimulation, the voltage stimulation appeared to be nearly comparable to that of the single electrode. In contrast to the effective depth of penetration, however, the paddle-array voltage stimulation consistently showed a far higher effective volume (about five times) than that of the single electrode.

In the current stimulation, the effective depth with the single electrode was notably higher than in the paddle-array current stimulation, while the single electrode current stimulation yielded slightly larger or comparable effective volumes to the paddle-array current stimulation. With respect to the amount of current regulated, the single electrode stimulation appeared to be more efficient than that of the paddle-array.

### Stimulation effects over computational models

**Figure 6:**
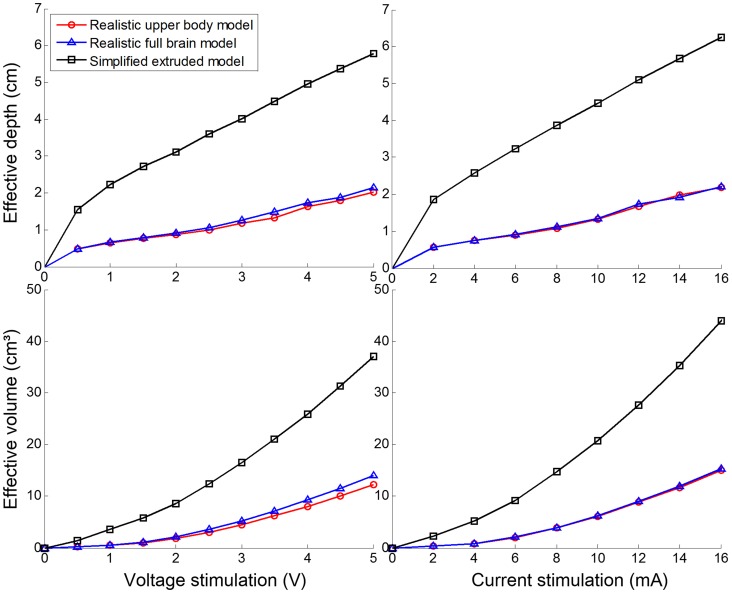
The influence of the geometry of the brain model on the effective depth and effective volume with stimulation voltage (V) and current (mA). Anisotropic conductivity in the white matter and a single electrode configuration were applied.

**Figure 7:**
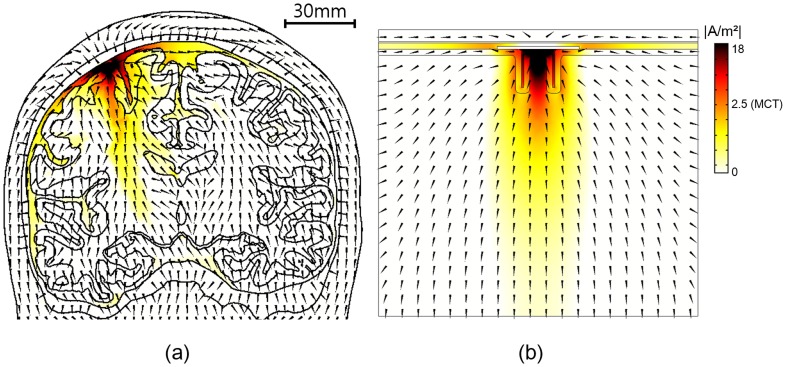
Current density distribution and flow in the brain. Realistic upper head (a) and simplified extruded slab model (b). The anisotropic conductivity in the white matter and a single electrode configuration were applied.

To investigate the influence of the geometry of the brain model in the simulation of SuCS, we developed 3 types of models: a realistic upper body model; a full brain-only model, and a simplified extruded slab model. For simplicity, the comparative investigation was performed with the single-electrode configuration and with anisotropic conductivity **(**normalized anisotropic conductivity**)**. Figure 6 shows the effective depth and volume of the three models as increasing stimulation voltage and current. In the two realistic models (upper body and full brain-alone models), there was no substantial difference in stimulation effects (effective penetration depth and volume), even though voltage stimulation effects appeared to be slightly higher in the full brain-only model than in the realistic upper body model. However, both the effective penetration depth and volume in the simplified extruded slab model were substantially higher than in the others. These substantial differences were observed in both voltage and current stimulations. Figure 7 illustrates the current density distribution and flow between the realistic upper body and simplified extruded slab models. The major feature of current density distribution appeared to be similar; current density under the electrode was highest and became gradually lower farther from the electrode. However, the current density in the simplified extruded slab model was distributed more widely and deeply than in the realistic upper body model. Furthermore, the predicted current flow in the extruded slab model was trivial due to its simple geometry and conductivity tensor. However, the current flow in the realistic upper body model was far more complicated due to the complex brain structure.

### Stimulation effects between white matter isotropy and anisotropy

**Figure 8:**
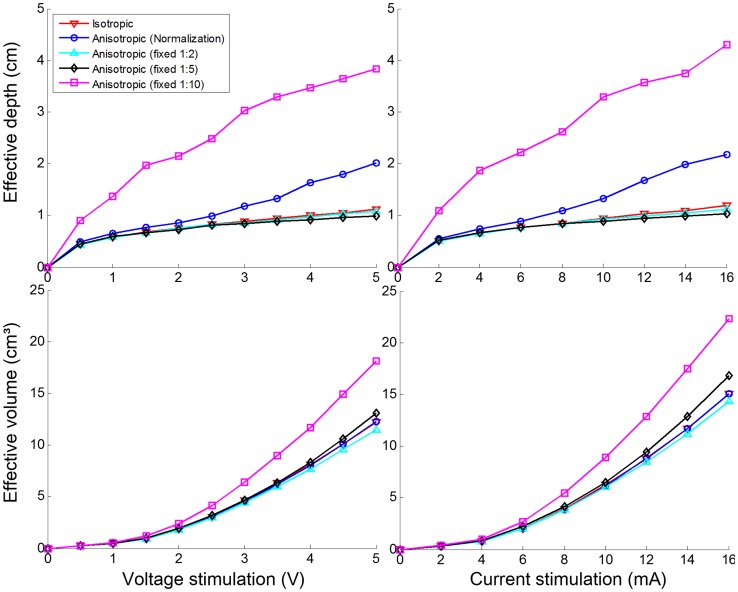
The influence of white matter anisotropy on the effective depth and effective volume of single electrode stimulation with stimulation voltage (V) and current (mA).

To investigate the effect of white matter anisotropy, we compared four types of anisotropy models (normalized conductivity and three fixed ratios: 1∶2, 1∶5 and 1∶10) and an isotropy white matter conductivity model. For simplicity, this comparative investigation was performed on the realistic upper body model with single electrode (voltage and current) stimulation. The effective penetration depth and volume for these isotropy and anisotropy conductivity models are illustrated in Figure 8. Both voltage and current stimulations were compared as well. The isotropic and anisotropic models with fixed ratios (1∶2 and 1∶5) showed similar trends and amplitudes in voltage and current stimulations. The anisotropic models with normalization and fixed ratio (1∶10), however, yielded a larger effective penetration depth. With respect to the effective volume, the fixed ratio (1∶10) anisotropy conductivity model yielded notably higher volume (for 2V or greater in voltage stimulation and for 6 mA or greater in current stimulation) than the others.

**Figure 9:**
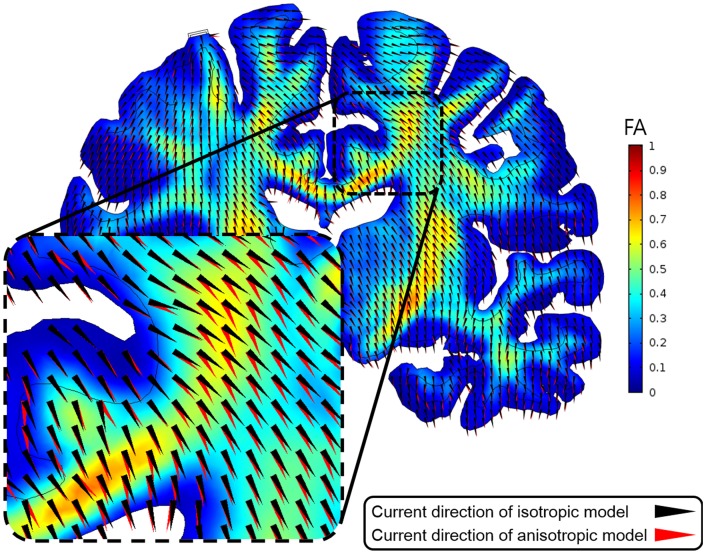
The coronal slice of the fractional anisotropy (FA) map and current direction for the isotropic and anisotropic models. A realistic upper body model and single electrode configuration were applied. Dotted area encircles the corpus callosum.

Figure 9 shows the distribution of fractional anisotropy (FA, which is defined in equation (4)) and direction of current flow for the isotropic and normalized anisotropic conductivity models. The FA value is usually higher in the white matter than gray matter, especially the corpus callosum and internal capsule. Around the stimulation electrode, the direction of current flow for the isotropic and anisotropic models was similar; in the dotted area, however, the difference in the direction of current flow was greater than around the electrode. Except for the area around the stimulation electrode, most of the current flowing in the white matter differed between the isotropic and anisotropic models; the current flow in the anisotropic model was guided primarily by conductivity tensor (the direction of fibers in the white matter). Thus, the anisotropic and isotropic models also had a different direction of current flow in the white matter. Further, these differences were likely to be greater at area with high FA values or when the current flow was far from the stimulation electrode, even in the gray matter, which has isotropic conductivity.

### Positioning of reference electrode

**Figure 10:**
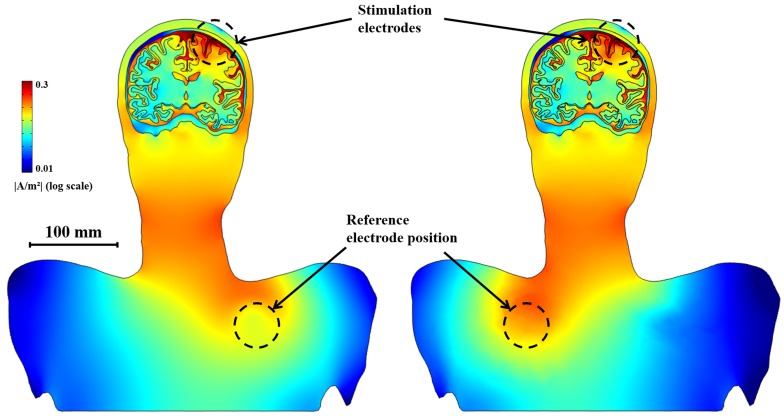
The spatial distribution of current density of 1V using right and left reference electrode model. The results in the brain are the same with the reference electrode on the right (left) and the left (right). However, the distribution of current density is flipped horizontally only under the neck.

The position of the anode or cathode electrode(s) and the reference electrode yielded slightly different distributions of the electric field in tDCS [13]. This led us to investigate the effect of reference electrode positioning in SuCS. In cortical stimulation, a reference electrode is usually implanted in the upper chest. Thus, two representative and different positions of the reference electrode may be considered: left and right sides of the chest. In our simulation, we found that there was no substantial difference in current density distribution in the brain between the two reference electrode positions (maximum relative difference (voltage) between two models considering difference of reference electrode position was approximately 6.7%; furthermore, the effective penetration depth and effective volume were nearly the same). However, we observed that the current density distributions around the neck and shoulder were flipped horizontally (Figure 10).

## Discussion

### Efficacy of cortical stimulation

Although it is not fully understood, invasive or noninvasive cortical stimulation is known to play a considerable role in increasing the plasticity or long-term potentiation that contributes to recovery from neurological disorders. Extensive research on TMS and tDCS has proposed several mechanisms by which plasticity is increased: diaschisis, peri-infarct reorganization, disinhibition of ipsilesional hemisphere, and the facilitation of the contralesional hemisphere [47], [48]. If ECS is combined with rehabilitative training versus rehabilitation alone, significant neurological recoveries have been observed in chronic stroke patients [3], [49]. Animal studies have also shown that cortical stimulation with rehabilitative training can enhance motor recovery in primates and rats [50]. It is known that surviving neurons near the area of the brain damage (penumbral zone) are engaged in compensating for lost functions [51]. Based on observations supporting the hypothesis that neurite growth and synaptogenesis in the neocortex correspond both spatially and temporally with behavioral recovery [52], the extent of electrical stimulation is likely to be critical in the reorganization of neural functions. Therefore, the determination of current density distribution in this work is clinically relevant to the prediction of the therapeutic efficacy of cortical stimulation.

### The effect of electrode configuration

In this work, two types of electrode configurations (paddle-array and single electrode) were evaluated. As expected, with voltage stimulation, the paddle-array using multiple electrodes had a broader and deeper effect than did the single electrode stimulation. At 5.0V, the paddle-array voltage stimulation was approximately 5.96 times higher in effective volume and approximately 1.96 times higher in effective depth. In the paddle-array, electrodes are distributed over the cortex, thereby stimulating a wider area; the volume of the cortex activated may increase proportionally to roughly the third power to the depth of cortex activated, thus, a 5.96 times higher effective volume is expected to yield an approximately 1.81 times greater effective depth. However, it yielded an effective depth approximately 1.96 times higher. Further, in isotropic models (not shown here), the paddle-array voltage stimulation was approximately 5.73 times higher in effective volume and 2.51 times higher in effective depth. Considering five electrodes (paddle-array) versus one electrode, such a difference is comparable to or more efficient than expected; this suggests that in the voltage stimulation, there is some synergistic effect of the multiple electrodes in the paddle-array.

In contrast, the paddle-array current stimulation seemed less efficient than that of the single electrode in terms of the effective penetration depth, and also yielded a slightly lower effective volume. This can be understood in that, due to the regulation of the amount of current, it could not be expected to produce the same synergistic effect as multiple electrodes.

Nevertheless, depending on the situation, paddle-array stimulation can have the following benefits: for stimulating deep areas of the brain, paddle-array electrodes may produce deep stimulation at lower voltages than single electrode stimulation; for example, as shown in Figure 4, the effective depth of penetration for the paddle-array at 2.5V is roughly equivalent to that of single electrode stimulation at 4.5V. Thus, it may reduce the side effects of stimulation (e.g., seizures) due to high voltage stimulation in the focal brain area. Moreover, the paddle-array can inherently stimulate a broader area of the brain than the single electrode; it was reported [3] that for extensive stimulation two sets of paddle-array electrodes were implanted epidurally on the motor/premotor cortex and Broca’s area. As shown in Figure 4, there was no substantial difference in the depth of penetration between the single electrode and paddle-array voltage stimulation when 2V or fewer was input. Thus, to see the benefits of the paddle-array voltage stimulation, an input voltage higher than 2V is required.

In addition to the disc electrode, other types–e.g., ring electrodes and covered discs [31], [53]–can be used in cortical stimulation. It has been reported that there is no significant difference between ring and disc electrodes for both SuCS and ECS [31]. However, ring electrodes can be used to accommodate the sensors in order to monitor neural responses to stimuli. Reference [30] compared unipolar and bipolar stimulations in the precentral gyrus model. They found that unipolar stimulation showed far greater effects than bipolar stimulation. From this study, it is expected that single-electrode voltage stimulation (unipolar) may be a reasonable choice, while paddle-array voltage stimulation (unipolar) can be an alternative when input voltage of 2.5V or higher is necessary.

### Influence of brain models

Depending upon brain model selection, the effect of stimulation may show a quantitative difference in terms of two efficiency measures. When considering isotropic or anisotropic brain models, the simplified extruded slab model has been thought to be a reasonable choice for the analysis of the local effects of SuCS. However, in our simulations of anisotropic and single electrode stimulation, the difference between the simplified slab model and realistic upper body model were considerable. As shown in Figure 6, the difference between the simplified extruded slab model and realistic upper body model was substantially great in both voltage and current stimulations. This indicates that the model mismatch may play a crucial role in estimating stimulation effects. Our proposed computational models are far more realistic than the simplified extruded slab model; however, it is not evident that our models yield more accurate stimulation effects than the simplified model due to the absence of ground truth. For this reason, a validation study with a realistic head phantom is under investigation.

Assuming that these simulation results are applied to predict the effects of stimulation among patients who plan to have electrodes implanted, it is expected from our investigation that the difference in stimulation effects (effective depth and volume) demonstrated between the simplified extruded slab model and the realistic upper body model may be significantly large due to the possible variability of individual cortical structures from in-born structure or brain damage. In the practice of cortical stimulation for clinical purposes, individual detailed brain modeling (personalized computational model) based on magnetic resonance imaging (MRI and DTI) with electrodes is strongly recommended to assess the accurate prediction of cortical stimulation effects.

Further, these differences between brain models would prove much higher in the analysis of neuronal responses (using neuron modeling or activating function). In the results of [26], neuronal responses elicited by cortical stimulation were very sensitive to the position of the stimulation electrodes, geometry, and polarity. Also, the investigation of neuronal responses is based on finite element analysis [23], [24], [26], [27], [43]. Thus, precise finite element analysis based on reasonable brain model selection should be conducted before analyzing neuronal response.

The difference in voltage stimulation between the realistic upper body and full brain-only models was quite small. As described, this small difference may be caused by different impedance between the stimulation and reference electrodes. This phenomenon decreased using impedance matching stimulation: current stimulation, as shown in Figure 6 (right). Thus, using a head model that includes the upper chest to apply reference electrode design seems to offer no benefits in current stimulation.

### Anisotropic conductivity in the white matter

To predict current density distribution in the white matter precisely, the accurate conductivity information is required; diffusion tensor image has been widely used to estimate conductivity tensor in the brain. Estimation of conductivity from diffusion tensor image is commonly done under the assumption that the eigenvector of diffusion tensor shares the eigenvector of conductivity tensor. Further, the determination of eigenvalues of conductivity tensor is required, but there is no clear justified approach on it. Despite this problem, the fixed ratio or normalized anisotropic conductivity models were introduced here, which is commonly used in brain modelling [54], [55]. Expectedly, the fixed ratio (1∶10) anisotropic conductivity yielded substantially higher effects of stimulation than others, which is evident that the effect of anisotropic conductivity may be not negligibly small, but it may play some role in estimating the current density in the cortical stimulation. For the more precise estimation of conductivity in the brain, in vivo measurement or other advanced techniques may be quite appealing.

### The implications of reference electrode positioning

In this work, a chest model was incorporated into the brain model to investigate the effect of the positioning (right or left) of the reference electrode; this is a more reasonable model considering that the reference electrode is implanted in the chest in unipolar SuCS. We found that the positioning of the reference electrode yielded no substantial change in the current density distribution in the brain. However, we observed a somewhat higher current density around the reference electrode as a result of the far higher conductivity of the reference electrode or stimulator devices. This may cause unwanted side effects. For example, among patients who have a pacemaker, implantation of the electrode in the left chest should definitely be avoided, as the electrode could cause the pacemaker to malfunction. Therefore, if the purpose of a study is to investigate the safety of the effects of unipolar stimulation on other organs, we recommend the use of the realistic upper body model.
